# Community Impact of Capacity-Building to Develop Trauma Resilient Communities

**DOI:** 10.1007/s10488-024-01429-4

**Published:** 2025-02-17

**Authors:** Todd P. Gilmer, Kimberly Center, Natalie J. Romero, Lila Burgos, Joelle Greene, Elizabeth Siantz, Lawrence A. Palinkas, Amy E. Lansing

**Affiliations:** 1 Herbert Wertheim School of Public Health and Human Longevity Science, University of California, San Diego, La Jolla, CA USA; 2 School of Social Work, College of Health and Human Services, San Diego State University, San Diego, CA USA; 3 UCLA Latino Policy and Politics Institute, Los Angeles, CA USA; 4 ORS Impact, Upland, CA USA; 5 College of Social Work, University of Utah, Salt Lake City, UT USA; 6 Department of Psychiatry, University of California, San Diego, La Jolla, CA USA; 7 Department of Sociology, San Diego State University, San Diego, CA USA

**Keywords:** Community capacity building, Trauma;, Community partnerships

## Abstract

This paper explores the impact of an initiative designed to promote trauma resilient communities by mitigating social determinants of health and reducing health disparities through capacity-building partnerships. Nine regional partnerships in Los Angeles County were funded to build community capacity to adapt to and recover from traumatic events through outreach and engagement with community members, training related to the impact of trauma, linkages of community members to existing services, and developing new services for target populations. Primary quantitative and qualitative data on community impact were gathered from agency leads (N = 10), partnership members (N = 136), and community members (N = 42). A convergent sequential mixed methods design (qual → QUAN → qual) was selected to provide both breadth and depth of understanding about the impact of community capacity-building from multiple perspectives. From January 2018 through July 2023, partnerships conducted over 30,000 community capacity-building activities with over 1.4 M community members and created 101,370 successful linkages to resources and services among 12,663 unique community members. Agency leads, partnership members, and community members converged on three main themes describing the initiative’s impact: (1) Building more empowered and resilient communities; (2) Normalizing help-seeking and reducing the stigma of mental health; and (3) Connections within partnerships/families and with community members. On average, 82% of partnership members endorsed medium to large positive changes in the impacts identified during qualitative analysis. Future work should examine whether contracting with community-based organizations is an effective approach for health systems to promote health equity.

## Background

Trauma is a significant public health issue, negatively impacting a range of health outcomes that disproportionately impact vulnerable populations (Hughes et al., 2017). Similar to populations worldwide, a large portion of the United States (US) population has experienced trauma or significant adversity, including life-threating trauma such as interpersonal violence and natural disasters, potentially traumatic events such as emotional abuse or non-violent loss, or exposure to poverty, historical trauma, systemic racism, or discrimination or disenfranchisement based on race/ethnicity, gender-identity, or sexual orientation (Felitti et al., 1998; Kessler et al., 2017; Sacks & Murphey, 2018). While the factors associated with traumatic exposures and trauma responses may be varied, they often share similar consequences, including diminished mental health, functional impairment and the amplification of health disparities, taxing already scarce or strained individual, social and community resources (Jaggi et al., 2016; Williams et al., 2018). Exposure to trauma simultaneously increases the level of a community’s needs while reducing trust in public health and social services (Egede & Walker, 2020).

Mental health administrators recognize the widespread experience of trauma in their communities and their limited ability to address community-wide trauma within their service systems (Loomis et al., 2019). In Los Angeles County, the Department of Mental Health funded nine regionally based, community-embedded partnerships with the goal of building capacity to address trauma within their communities (Gilmer et al., 2021). These partnerships implemented community capacity-building strategies that focused on specific populations such as parents of young children, transition-age youth, justice involved populations, and multigenerational families. Each strategy involved community outreach, community events, group activities with community members, trainings in the community, and linkages to resources among community members. Collectively, these strategies were designed to facilitate capacity-building goals by addressing the consequences of trauma across the lifespan and increasing awareness of, and access to, resources and supports for vulnerable and underserved groups within their own communities.

Although there exist well developed frameworks for trauma-informed care, there is less guidance available for developing trauma-resilient communities (Substance Abuse & Mental Health Services Administration, 2014a, 2014b). Trauma-informed care is an approach to delivering mental health services that is sensitive to the social, psychological and biological consequences of trauma, with an increased eye towards cultural sensitivity and humility (Substance Abuse & Mental Health Services Administration, 2014). Trauma-informed organizations use the principles and practices of trauma-informed care to reduce the impact of trauma through culturally competent trust-building, safety, transparency, empowerment and collaboration which promote trauma resilience. Resilience is process of successfully adapting to challenging life circumstances; thus, resilience to trauma refers to an individual’s ability to cope and move forward after a traumatic experience while community resilience is the ability of a community to adapt and recover from traumatic events (Freitag et al., 2014).

Previous efforts at developing system or community capacity to address trauma have focused on education and training in the delivery of trauma-informed care and developing trauma-informed partnerships (Champine et al., 2022; Loomis et al., 2019; Matlin et al., 2019; Tebes et al., 2019). These efforts have focused on specific populations or service systems, and their reports have been limited to descriptions of the process of implementation (Champine et al., 2022; Loomis et al., 2019; Matlin et al., 2019; Tebes et al., 2019). In contrast, in this paper we examine the community impact of a capacity building initiative that spans multiple populations at risk for trauma, using diverse, multi-level strategies to address trauma and build community resiliency across several regions of the most populous county in the US.

This study is part of a larger, longitudinal, mixed methods evaluation of this capacity-building initiative. Previous work quantified the efforts at capacity building by partnerships, linkages to resources, and improvements in coping and sense of community connectedness among community members engaged in services over time (Gilmer et al., 2021). Trust was established and sustained through community engagement and cultivating relationships with community members; embodying core values of trustworthiness; and sharing decision-making, championing autonomy, and addressing barriers to trust (Lansing et al., 2023). The aim of the present study was to identify the community impact of these capacity-building efforts from multiple perspectives using mixed methods that provide both breadth and depth of understanding.

## Methods

### Design

**Fig. 1:**
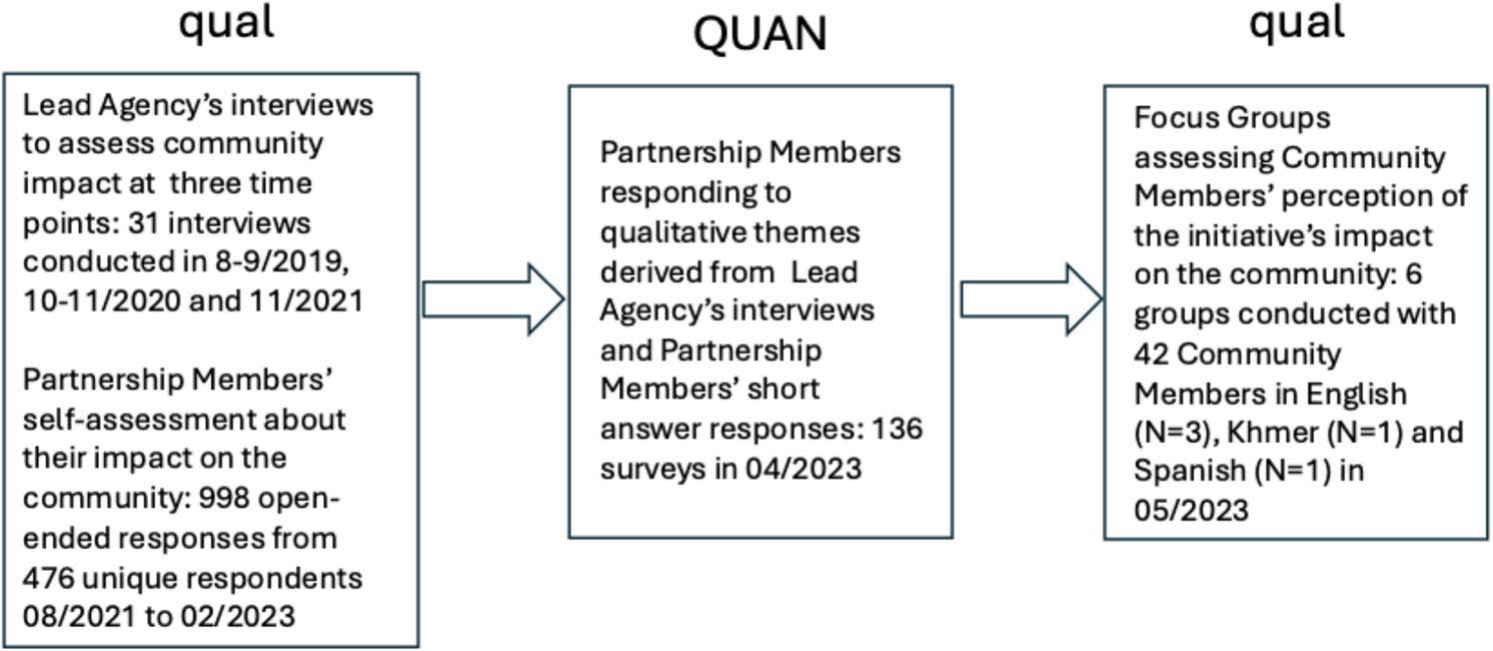
Mixed method data collection to assess community impact

This paper employs qualitative and quantitative data to evaluate the community impact of capacity-building to address trauma from three perspectives: those of agency leads (i.e. those leading the partnerships), partnership members, and community members. This paper employs a sequential mixed method study design of qual → QUAN → qual (see Fig. 1) (Palinkas et al., 2011). The perspectives of agency leads were captured qualitatively through three waves of longitudinal individual interviews, and the perspectives of staff from community-based partnerships were captured qualitatively through short answers to open-ended questions on four waves of surveys. These two sets of qualitative data were combined to develop a set of quantitative Likert response questions that were administered to all partnership members in a separate cross-sectional survey. The perspectives of community members were captured qualitatively using focus groups. This mixed methods design achieved triangulation (convergence) across three data sources (Creswell & Plano, 2018). The qualitative data from the agency leads and partnership members provided a depth of understanding while the quantitative data from partnership members provided breadth (i.e. complementarity). Qualitative data from the focus groups were then used to elaborate on the quantitative data by describing how the initiative was experienced by community members (i.e. expansion).

### Study Samples

Qualitative interviews were conducted with agency leads from nine community partnerships. Agency leads were key informants knowledgeable about their agency, partnership, and strategies, and were instrumental in decision-making and setting the tone for their partnership collaborations. Most partnerships were represented by a single person unless a partnership had separate leads representing different strategies or distributed duties. In cases with more than one agency lead, interviews were conducted separately. All agency leads agreed to participate in the interviews. Demographics for the agency leads were previously reported by year (Lansing et al., 2023) with 10–11 interviews conducted annually over 3 years, with most agencies leads remaining in their positions and one to two partnerships having two leads. Across the three waves, on average, 80% of interviewees were female with the following race/ethnicity composition: 29% Black; 36% Latinx; 23% non-Latinx White, and 10% each Asian and Biracial/multi-ethnic. A total of 31 semi-structured interviews were conducted with agency leads at three timepoints to coincide with (a) the end of the first year of initiative implementation (baseline), conducted in person in August and September of 2019; (b) the end of the second year (mid-point), conducted in October and November of 2020 via Zoom; and (c) the end of the third year (final), conducted in November 2021 via Zoom.

Over the course of the initiative, from January 2018 through July 2023, the nine regionally based community partnerships involved 1153 unique partnership members from 121 community-based organizations. Partnership members were queried about the most important impact of their work on the community at four semi-annual timepoints (from August 2021 through February 2023). A total of 998 short answer responses to this open-ended question were provided by 476 unique partnership members.

A cross-sectional survey with questions developed from the agency lead interviews and partnership-wide short answers was fielded in April 2023. At this time, the nine partnerships included 542 active members from 73 community-based organizations. A total of 136 members (25%) responded to the survey: 12 (9%) were executive or leadership roles; 33 (24%) were in administrative roles including program managers or coordinators; 13 (10%) were in clinical or specialist roles; 71 (52%) were community ambassadors or front-line staff; and 7 (5%) were other partners, consultants, or subcontractors. Those in leadership roles (60%) were most likely to respond to the survey followed by community ambassadors (28%) and those in administrative or clinical/specialist roles or other roles (20%).

Purposeful sampling was used to recruit six focus groups that represented the diversity of community members’ cultural backgrounds and a range of capacity building strategies. A total of 42 community members were recruited by three partnerships to participate in groups conducted in three languages: English (N = 3), Khmer (N = 1), Spanish (N = 1), and Spanish with simultaneous English translation (N = 1). Focus groups included community members from the four most common capacity building target populations: caregivers with young children ages 0–5 who have experienced trauma and/or are at risk for trauma; transition-age youth ages 16–25 years old who were at risk of and/or experiencing mental illness or homelessness; youth, adults, and older adults with histories of incarcerations or juvenile justice system involvement; and multigenerational families who have experienced intergenerational trauma. Three focus groups were conducted in person and three were conducted via Zoom, all in May 2023. Focus group sessions lasted between 75 and 120 min.

### Data Collection

All methods were carried out in accordance with relevant guidelines and regulations and were reviewed and approved by the University of California, San Diego Institutional Review Board (IRB #201892X). The data presented in this paper were previously collected as part of an evaluation of a program aiming to support partnerships in building community capacity to access resources and offset the impact of community trauma. The UCSD IRB determined that (a) the secondary use of the data for research purposes presented no more than minimal risk to human subjects; (b) the study qualified for review through the expedited procedure; and (c) it qualified for a waiver of informed consent.

#### Community Capacity-Building Activities

Data on community capacity-building activities are presented to provide context for community impact. Community outreach, community events, group activities in the community, and training in the community were documented by partnership staff using registry-based tracking systems. An Event Tracker was used to collect the name, date, and type of each event, and the number of people who attended. These data were used to quantify the types and numbers of capacity-building activities and the numbers of participating community members. A Referral and Linkage Tracker was used to document referrals made for specific individuals and tracked whether the linkage was successful, unsuccessful, or in progress. Data from the Referral and Linkage Tracker was used to quantify the number and types of successful linkages.

#### Agency Lead Interviews

The longitudinal lead agency interviews addressed a range of evaluation questions (Lansing et al., 2023). The baseline interview assessed key aspects of implementation and adaptations during the early phases of this learning initiative, as well as agency leads' understanding about trauma exposures and trauma-related needs in their respective communities. The midpoint interview explored themes from the baseline interview, assessed COVID-19 related adaptations, and addressed emerging impacts such as how trauma-informed approaches facilitated community capacity building efforts. The final interview followed up on themes related to specific aspects of trauma-informed practices that emerged as essential during the initiative (e.g., safety, cultural sensitivity); explored the impact of other approaches used during the initiative (e.g. learning-based project culture, community resiliency models) and challenges faced by all partnerships (e.g., the pandemic); and queried sustainability goals.

This paper focuses on three waves of interview data related to the impact of trauma informed approaches to community capacity-building on the community. Interview questions included: “How do you think your strategy/strategies contribute to increasing your community’s ability and willingness to support its members in ways that reduce the impact of trauma?”; “Has [the initiative] opened up your community’s conversations about trauma, adversity and the health impact of these experiences?”; and, “In what ways have your community members grown in how they express, discuss, think about or respond to adversity (trauma), and how adversity impacts their lives?” Interviews were conducted by MA and PhD trained members of the evaluation team who were familiar with the overall project goals and were experienced interviewers. Interviews lasted approximately 90 min and were professionally transcribed for analysis using the audio and video recording from Zoom.

#### Partnership Surveys

Partnership members were queried semi-annually throughout the initiative regarding opportunities for training in trauma-informed practices. This survey was revised in 2021 to explore partners perspectives on the impacts or legacy of community capacity-building by including an open-ended question: “What do you think will be the most important outcome (the greatest impact or lasting legacy) of [the capacity building initiative] for your community?”.

Responses submitted in Spanish were translated into English. Responses to the open-ended questions were reviewed and organized into a summary template, which organized the impacts by themes or domains.

The themes/domains identified in the short answer responses were compared to those that emerged from agency lead interviews. Three core themes emerged and overlapped from the longitudinal qualitative interviews and open-ended responses: *Building more empowered and resilient communities*; *Normalizing help-seeking and reducing the stigma related to mental health*; and *Connections with the community*. A new cross-sectional retrospective survey was developed to quantify these three core themes. The survey included twenty specific community impacts related to the themes. These impacts were derived primarily from responses to the open-ended questions with confirmation from the agency lead interviews.

#### Community Focus Groups

Focus group questions were crafted to address community members’ experiences of the initiative and were aligned with the three core themes identified in the previous, longitudinal qualitative work (i.e., three agency lead interviews and short answers from four partnership surveys). Focus groups were conducted using Ripple Effects Mapping (REM). REM is a highly visual, reflective process that has been used to evaluate a range of participatory programs in a variety of settings (Kollock et al., 2012). REM is particularly well-aligned with the goal of linking partnership activities to impacts in the community and is thus likely to yield a greater depth of reflection, and more active and collaborative participant engagement, than would have resulted if we had used a traditional focus group approach. We adapted the process outlined by Welborn et al. (2016) for both in-person and virtual forums (Welborn et al., 2016). Since focus group participants were not fully aware of, nor had participated in, all the activities provided as part of the partnerships’ efforts at community capacity building, we began each session by providing a list of activities (created in collaboration with partnership staff) so that participants could more easily recall the activities and services they experienced and recognize that other session participants might have had different experiences.

The group then moved into the mapping process. Community members were encouraged to think about the impact the activities had on themselves, their family and friends, and their community. The first ripple of the map was developed based on the questions: “Which [agency name] events or groups have you participated in?” and “Because of these events or groups, what are you or the other participants doing differently?” Follow up questions included: “What unexpected things have happened during your involvement with [agency name]?”, and “What connections with or between others have you observed as a result of your involvement with [agency name]?” The second ripple was developed based on the question, “How have these activities affected your family and friends?” The third ripple was developed using the question “What changes are you seeing in your community?” For virtual sessions, community members were invited to share in the chat or aloud with the group. Participants then mapped those impacts and experiences using post-it notes for in-person and virtual sticky notes on Jamboard for online sessions. Focus groups were recorded and transcribed verbatim; transcripts for sessions conducted in Spanish and Khmer were professionally translated into English.

### Data Analysis

#### Quantifying Community Capacity-Building Activities

We used data from the Event Tracker to quantify the types and numbers of capacity-building activities and the numbers of participating community members. We mapped specific activities to four major types of events: (1) Community outreach includes efforts at engaging community members and organizations; (2) Community events involve small to large groups in one-time activities; (3) Group activities in the community include small groups of community members in recurring activities; and (4) Trainings in the community include community-based training in trauma-informed principles and other subjects related to public and community health. We calculated the numbers of events and community members’ participation in events. We used data from the Referral and Linkage Tracker to calculate the number and distribution of linkages by type of resource or service.

#### Qualitative Analysis of Lead Agency Interviews

We employed a Grounded Theory methodology, incorporating iterative coding processes for all interviews. MAXQDA Plus software was used for coding (VERBI Software, 2020) (Charmaz, 2015; Glaser & Strauss, 2017) and reliability was 88.7% among coders. First Cycle Coding Elemental Methods included *Initial*, In Vivo, and *Descriptive Coding*, as well as *Focused Coding* to highlight question-driven and organically emerging categories. *Memo Coding* was used in the form of written comments, impressions and ideas shared among team members, both within and outside the MAXQDA environment (Saldaña, 2021). *Axial Coding* is usually considered ‘*Second Cycle*’, but interview questions and probes were also constructed to examine variation occurring across and among agencies, partnerships, and their communities. After each interview wave, the codebook was expanded to include new codes as needed and coding decision rules were updated to improve reliability and to provide an audit trail.

*Process Coding* was used to operationalize and classify key themes and findings related the impact of community capacity building on community members. Themes were created initially by the coders and supervisor, then reviewed and further developed by the larger evaluation team. Theme development included (1) examining within and across question themes, (2) general across interview themes, (3) individual agency leads responses within timepoints and across interview waves, and (4) across agency lead responses within and across timepoints. We used data-based, inductive Values Coding to capture values, beliefs and attitudes reflecting agency leads’ worldviews from their own (i.e., “emic”) perspective (Saldaña, 2021). Exemplary quote selection was iterative; for example, independent selections were followed by comparison and consensus-building among multiple team members.

#### Quantitative Analysis of Partnership Surveys

As described above, twenty partnership survey questions were developed to quantify specific impacts related to three core themes that were aligned between the lead agency interviews and open-ended questions from partnership members: *Building more empowered and resilient communities* (12 questions, Q = 12); *Normalizing help-seeking and reducing the stigma related to mental health* (Q = 4); and *Connections with the community* (Q = 4). Each question queried whether the respondent recognized a specific change in the community. Likert scale response options reflected a 0–4 scale, with higher scores reflecting larger changes: “I’m not sure” (0), “No change” (1), “Small change” (2), “Medium change” (3), and “Large change” (4). The reliability of the 20 items was high, with an inter-item scale reliability coefficient of 0.97 overall, and 0.97 for Building more empowered and resilient communities, 0.93 for Normalizing help-seeking and reducing the stigma related to mental health, and 0.96 for Connections with the community. We calculated the frequency responses to each question. For each theme, we identified the impact for which most respondents indicated a “Large change,” as the most highly endorsed impacts may help to identify where the initiative was most effective.

#### Qualitative Analysis of Community Focus Groups

Consistent with the Ripple Effects Mapping (REM) approach, data from the focus groups were analyzed using a template approach that explored how the initiative impacted study participants, their friends and families, and their communities (Hamilton and Finley, 2019). This involved organizing the data in a summary template that explored how program participation impacted these three levels of participant experience. These domains were identified a priori to align with the REM data collection process. Two members of the study team read and coded each transcript with attention to these domains, and discrepancies in codes assigned were resolved through consensus. Each coded transcript was then entered into Dedoose qualitative software. Coding reports were prepared that captured each of the three levels of participant experience, which were then read and summarized. Qualitative results derived from focus group transcripts were then compared to the REM maps, to ensure alignment between these two types of data.

## Results

A summary of community capacity-building activities under the initiative is provided in Table 1. As of July 2023, the 9 partnerships conducted over 30,000 community capacity-building activities with over 1.4 M community members. Community capacity–building activities included community outreach, community events, group activities in the community, and trainings for community members. Community outreach was intended to connect with large numbers of community members and increase awareness of available resources and supports and reached over 500,000 community members. Community events were intended to cultivate relationships and build trust with community members, also reaching over 500,000 community members. Group activities were designed to foster social connectedness and resilience among target groups and reached over 155,000 community members. Trainings in the community were meant to build awareness of trauma and its impact in the community and reached nearly 257,000 community members. Partnerships established 101,370 successful linkages to resources and services among 12,663 unique community members (see Appendix Table). There was an average of 8.0 linkages per community member over the course of the initiative. The most common service linkages made were for food distribution (42%), education (10%), clothing (9%), housing (7%), and health care (6%).

**Table 1:** Community Capacity Building Activities from January 2018 through July 2023

Type of event	Number of events	Attendees per event, mean (SE)	Number of attendees	Examples
Community outreach	8270	61 (138)	502,856	Attendance at neighborhood meetings, meal delivery at motels and drop-in centers, mobile showers and laundry for homeless youth, and cultivating relationships with local businesses, administrators and educators at schools, community leaders and other community organizations to grow the partnership’s resource network
Community events	4184	124 (226)	516,833	Family fun nights providing a dinner and music or family friendly arts and crafts, BBQs in the park, drive-up back to school events where children received backpacks with virtual learning supports such as headphones, drop-in events for youth, and mindfulness hangouts
Group activities in the community	9929	16 (32)	155,320	Parenting classes to increase knowledge of child development, play groups for children to support healthy attachments, knitting and storytelling circles for intergenerational families who have experienced trauma, yoga and Zumba classes to promote wellness, and skills trainings to support individuals who are seeking employment or education
Trainings in the community	7630	34 (191)	256,669	Anger management, addressing domestic violence, developing a trauma-informed curriculum in the public school system, mindfulness practices for educators and school staff, strategies for identifying and engaging with someone who experiences a mental health crisis within the law enforcement or court system, COVID-19, racial injustice and health disparities, the use of technology to connect virtually, and taking care of mental health during extreme times of stress

Agency leads and partnership members concurred on three main themes involving the impact of the initiative on community and community members. Each theme is explored in more detail below. Exemplary quotes related to these themes are shown in Table 2. Partnership members’ endorsements of the proposed community impacts of capacity building are shown in Table 3. On average, 54% partnership members endorsed the impacts identified in the qualitative analysis as having a large change, 28% identified a medium change, and 8% identified a small change, 3% identified no change, and 7% were not sure.

**Table 2:** Exemplary quotes from agency leads’ perspectives on community impacts

Community impacts theme	Community impacts subtheme	Exemplary quote
Building more resilient and empowered communities	Empowering community members	I think one thing that we’ve noticed is an increasing number of our referrals are actually coming from current members, so they are starting to find their own voice. They’re finding the value in what they’re learning about trauma and resilience from us and they’re sharing with others. One of our clients her mom has a younger child and she was able to talk to her about why it’s so important to learn about this type of stuff and the mom is now a client of [the partnership]. So we found that some of our families are starting to refer others. They’re starting to feel more empowered with sharing information
	Increasing knowledge and skills regarding trauma	I think the trainings that we have been able to provide to the community have really helped them understand what that word [trauma] means. I think it’s thrown around a lot, but there’s not a clear understanding of like what trauma is and how that can be different things for different people. So, I think parents are definitely more comfortable talking about trauma and traumatic experiences and difficult experiences that they may not have understood to be trauma before this, but now they understand maybe their reactions to certain things with their children and why they parent the way that they do. So, I think that’s opened up a lot of conversations and then as far as just like in general having that vocabulary shared with like teachers and childcare providers, I don’t think that they’ve historically gotten that type of training. So, I think it’s opened up a lot of conversations
Normalizing help-seeking and reducing the stigma related to mental health	Normalizing help-seeking	… we had running groups, doing creative programming, doing the mindfulness hikes, doing the knitting group, were all folks that use these mechanisms and these avenues to connect people with resources. …what I'm referring to is demystifying and destigmatizing being engaged in services, being in or seeking out an EBT card, seeking out a rental assistance program, and because we've normalized asking for help, we've seen that across the board where people have been more comfortable asking for help. Sometimes I get calls from participants, and they're like, "I normally would have never called you, [name], but it seems like–", it seems like, you know, like in Spanish so I'm trying to translate it in my head, but like, "You're approachable, and we heard you talk at the last group, and I have a question, I have a son and they have.." And so just even the fact that I'm getting some of those calls in addition to [name] and everyone else is really evidenced by the fact that people are looking at– their help seeking behavior has changed because in this bubble of Innovation we've normalized that, that's part of our agreement is that we will ask for help, and we will try to get you the help, and then you bring in the [staff] members who are even more responsive and have the lived experience and are reinforcing the normalization of asking for help…
	Reducing the stigma related to mental health	It reduces the stigma because others get to hear from each other that I'm not the only one. You know, I'm not the only one whose husband just lost their job, who's struggling to put food on the table, who may for the first time be seeking, you know, being in line at a food bank. Like there's stigma around that sometimes. And seeking mental health services, too. And I think it just normalizes it when you hear from others, "Oh, she's going through the same thing," or, "He's going through the same thing. That family's experiencing what I'm going through." I think it just helps reduce that stigma or that fear of feeling like I'm the only one. It builds some connection and then I think allows for folks to then say, you know, I need to get connected to resources and my family needs, you know,– I really can't do this alone anymore. I need support from a professional. I need mental health services. I need to get a referral. Or I need to get connected to X, Y and Z, whether it's, you know, a food pantry or where to go to get, you know, I don't know, housing assistance or where to go to get tested for COVID. So I think just hearing from the experiences of others has allowed a space for others to then open up and has reduced some of the stigma around these experiences so that they feel like it's a shared experience, I think, and then they're able to seek those services that they need
Connections with the community	Connections with the community	We have a program at [partnership]—Children Exposed to Violence—and a lot of our [staff], when they hear about this program, they’re like, “Where was this when I was little? I was exposed to community violence, gun violence, gang violence. Why wasn’t this offered before?” So them knowing that it’s a tool, a resource that they would have utilized in their youth makes them want to take that out into the community more and reach the youth that these are available for you or your family—that they are available, at no cost to you. So at least that’s something that we’re all able to share, is our lived experience. Whether it's here in the East Side, whether it's in South L.A., we all have that experience growing up in L.A. of witnessing violence of some sort, so we’re able to relate to that, and we’re able to just have an open, safe space with each other

**Table 3:** Partnership members’ perspectives of community impacts of capacity building

	Not sure (%)	No change (%)	Small change (%)	Medium change (%)	Large change (%)
Building more empowered and resilient communities					
Community members …					
*Empowering community leaders*					
… are better able to advocate for themselves	7	4	6	26	58
… are better able to advocate for their communities	7	3	10	32	48
… have become leaders in their communities	10	4	13	32	40
… share awareness of community resources and supports more	5	3	4	31	57
… feel more connected within their community through group activities and community events	7	3	7	29	54
… use their knowledge gained to help their communities	6	4	11	26	53
*Increasing knowledge and skills regarding trauma and community resiliency*					
… have a better understanding of the impact of trauma on health and wellbeing	8	3	8	29	52
… have a better understanding on the impact of trauma on children or youth	5	4	8	26	57
… have a better understanding of how trauma can be passed down through generations	8	4	6	30	52
… have a common language to talk about trauma in their community	7	4	7	38	44
… have learned new ways to deal with past trauma to make healthier decisions for their future	8	4	7	30	51
… use the coping skills and strategies they learned to manage their stresses and be resilient in the face of challenges	7	3	7	29	54
Normalizing help-seeking and reducing the stigma related to mental health					
*Normalizing help-seeking*					
… are more likely to seek out help when they need it	7	3	8	26	55
… are more likely to connect to services through linkages and referrals	6	3	9	24	58
*Reducing the stigma related to mental health*					
… are more comfortable discussing mental health issues that impact their lives and/or the lives of their family and loved ones	6	2	11	26	54
… trust our organization and feel comfortable (or safe) telling us what they need	5	4	8	28	55
Connections with the community					
My organization …					
… is more engaged within the community	6	3	7	24	61
… invites community members to have a voice in directing our activities	8	5	7	26	54
… has built trust with the community	7	3	4	25	61
… has built authentic relationships with community members	6	3	8	24	60

### Building More Empowered and Resilient Communities

The initiative’s work to build more empowered and resilient communities was the result of significant outreach and engagement of community members, linkages of community members to existing services, and services developed for specific target populations. Two subthemes were identified: empowering community members and increasing knowledge and skills regarding the consequences of trauma exposures. Outreach, engagement, and service delivery resulted in empowering community members to be better advocates for themselves and for their communities, and developing leaders who use their knowledge gained to improve their communities. Agency leads noticed that over time an increasing number of referrals came from community members, who were sharing what they had learned about trauma and resilience with other community members who they thought may benefit from the services provided by the partnership. The most highly endorsed impact of empowering community leaders was “community members are better able to advocate for themselves”, where 58% identified a large change.

Extensive community training in trauma-informed practices resulted in increasing knowledge and skills regarding trauma among community members, who now had a better understanding of the impact of trauma on health and well-being and a common language to talk about trauma in their community. Agency leads found that community members were more comfortable talking about trauma and difficult experiences that they may not have previously understood to be trauma, and were more aware about how past trauma affected their reactions to triggering events including parenting. The most highly endorsed impact of increasing knowledge and skills regarding trauma was “community members have a better understanding of the impact of trauma on children or youth”, where 57% identified a large change.

### Normalizing Help-Seeking and Reducing the Stigma Related to Mental Health

Partnership activities also helped to normalize help-seeking and reduce the stigma related to mental health. Agency leads found that outreach, engagement and group activities helped to build rapport between partnership members and community members and provided new avenues for community members to engage in services when needed. Community training in trauma informed practices and sharing of experiences among community members helped to reduce the stigma of mental health and mental health services. The most highly endorsed impact related to normalizing help-seeking was “community members are more likely to connect to services through linkages and referrals,” where 58% identified a large change. The most highly endorsed impact related to reducing the stigma related to mental health was “community members trust our organization and feel comfortable (or safe) telling us what they need,” where 55% identified a large change.

### Connections with the Community

The initiative served to strengthen connections with community members. Agency leads described working more collaboratively with community members, enabling community members to exercise their voice in directing activities, and building trust and authentic relationships with community members. Partnership staff with lived experience recognized the value of the services provided and how to connect community members to services. The most highly endorsed impacts of connections with the community were “my organization is more engaged with the community” and “my organization has built trust with the community”, where 61% (each) identified a large change.

### Impact of Capacity Building from the Perspectives of Community Members

Community members echoed the themes described above when queried about the impact of the initiative on themselves, their family, and their communities. Exemplary quotes related to these themes are shown in Table 4. When queried on the impact of the initiative on themselves, community members discussed skill building and resulting empowerment, and the normalization of their experiences within the community. Community members described how the partnerships supported skill building in areas such as selfcare and anger management, and how acquisition of those skills alleviated anxieties and challenges associated with parenthood. Participants valued the therapeutic benefits of program participation that occurred in supportive group environments within the cultural contexts of their specific communities, which left them feeling more empowered: “*to do what I needed to do.”* Participants described having improved skills for navigating personal relationships, increased patience with their children, and among participants in one focus group, reduced use of drugs and alcohol. Participation also normalized challenging experiences related to immigration and childrearing, among others, and decreased experiences of discrimination or public stigma.

**Table 4:** Exemplary quotes from community members’ perspectives on community impacts

Community impacts theme	Community impacts subtheme	Exemplary quote
Building more resilient and empowered communities	Empowering community members	Yes, I believe I have connected with others because I joined several workshops on autism, which helped me learn how to identify symptoms. This empowered me to take the initiative; I learned who to approach, and I could discuss it with my pediatrician. Once I figured out those symptoms, I could finally contact the Regional Center
	Increasing knowledge and skills regarding trauma	I was speaking about a story that I have encountered with my siblings and their issues at school in terms of bullying. I think I've learned a lot through these trainings. It's definitely skills that I can incorporate in my own family and definitely how I would respond to those issues.
Normalizing challenging experiences and reducing discrimination related to mental health	Normalizing challenging experiences	It makes me feel mentally and emotionally connected and more safe to be like, "Oh, I'm not alone. It's not just me in the room."
	Reducing discrimination related to mental health	When in attendance and there is mutual forgiveness. If something is wrong, there is no blame, no discrimination, then the friends are also no discrimination. United together and love each other. Yes, and it relieves the fears
Connections with the community	Connections with the community	I've felt like I'm part of a community, I am part of this family because of what we've cultivated within our community members here Our Cambodian refugees move here and some don’t know the languages…[are] illiterate, can’t drive, and they are still [at] home. They are sad but when they join in the ZOOM; they meet each other; they feel happy and … now can use telephone. Now they can login ZOOM, check the telephone, check the Facebook…

When queried on the impact of the initiative on family members, community members described a “*chain reaction,”* whereby their own participation had multiplicative benefits within their families, especially for their children. One participant described how participating in mental health workshops helped her to help her daughters navigate their own developmental stages. Other participants described their appreciation for a program which provided teens with opportunities for age-appropriate socialization and for a reading program that supported younger children in developing social skills.

When queried on the impact of the initiative on the community, many community members noted increased connections with the community and an increased sense of community that resulted from the initiative. Participants in community-based activities described how these efforts brought together and helped to foster leadership among community members. A Cambodian focus group participant described the importance of the initiative in creating connections within community for refugees and recently arrived immigrants including using ZOOM and social media to connect when in-person meetings were limited due to the pandemic.

## Discussion

This capacity-building initiative was able to provide outreach to and engage community members in activities designed to build resiliency to trauma. Capacity-building activities were extensive and included attending neighborhood meetings and cultivating relationships with community organizations, community events such as family fun nights and BBQs in the park, and group activities in the community such as parenting classes and storytelling circles. Trainings in the community fostered practical skills such as anger management and managing mental health in times of extreme stress. Community members received linkages to a range of services including food distribution, education, clothing, housing, and health care, that may offset health disparities over time.

Agency leads, community-based partnership members, and community members converged on three main themes highlighting the key impacts of the initiative: Building more empowered and resilient communities; Normalizing help-seeking and reducing the stigma of mental health; and Connections within families as well between partnerships and community members. On average, 82% partnership members endorsed these impacts, initially identified in the qualitative analysis, as representing medium to large retrospectively observed changes in their communities. Understandably, there were some differences in the descriptive language used among agency leads and community-based partnership members as compared to community members. For example, both agency leads and partnership members described normalization of help-seeking and reductions in stigma, while community members described this as normalization of challenging experiences and reductions in discrimination. Overall, the qualitative findings were consistent across agency leads, partnership staff and community members and convergent with the quantitative survey data.

Notably, healthy connections have been found to be associated with well-being and resiliency, particularly in the face of adversity, and a sense of belonging and collaboration have been identified as two of the key domains underlying community capacity building (Allen et al., 2021; Birgel et al., 2023; Gil-Rivas & Kilmer, 2016). Further, community-based collaborative approaches and networks have been found to facilitate the exchange of trauma informed resources and best practices, while improving public health (Ellis & Dietz, 2017; Matlin et al., 2019; Purington et al., 2020). The present initiative focused on engaging community members to be informed about the consequences of trauma and trained in skills or practices to enhance resiliency in the face of trauma. This approach, in turn, fostered stronger connections within families and between community-based partnerships and members of their broader communities. Destigmatization, reductions in perceived discrimination and normalization of challenging experiences, all likely contribute to enhanced cohesion and a sense of belonging and have the potential to “foster a culture of health” over time (Clair et al., 2016). Considered in this larger context, facilitating access to knowledge about trauma and its consequences, while improving access to needed resources, is not only empowering but appears to enhance belonging while breaking down barriers creates an interconnected sense of community that includes partnership and community members.

The approach to building partnership by the lead agencies in this study was consistent an approach to developing trauma-informed organizations including a commitment to being trauma-informed, creating infrastructure to support changes, building collaborations and involving key stakeholders including community members with histories of trauma, and continuous quality improvement (Substance Abuse and Mental Health Services Administration, 2014a, 2014b). A key addition to this framework is building capacity in the community to sustain improvements through empowerment, normalizing help-seeking, and building community connections. This focus on community resiliency, or the ability of the community to adapt to and move on from traumatic events, is consistent with efforts to address community preparedness for natural disasters. In their study of earthquake risks in three Washington State communities, Freitag et al. (2014) find that community participants identified social capital, including increasing community connections and building strong community organizations, as the most important factor affecting the community capacity to adapt to natural disasters (Freitag et al., 2014). This capacity is needed to support the identification and distribution of available resources after a severe natural event. Similarly, in their review of strategies to increase preparedness to deliver mental health services in response to impacts from climate change, Palinkas et al. (2020) recommend developing and implementing programs and policies to monitor and treat mental health problems, strengthening individual and community resilience, training community health workers, and assessments of risks and resources (Palinkas et al., 2020). Consistent in these approaches is the need for community empowerment and skill building, normalizing interactions with community organizations, and increasing community connections. The work presented here can help inform a standardized framework of community capacity building to build resiliency to future changes.

Currently, there is significant interest in addressing health equity specifically in partnership with health care systems. For example, the Advancing Health Equity initiative funded the Robert Wood Johnson Foundation aims to engage state Medicaid agencies and Medicaid managed care plans in aligning payment and delivery system reforms to reduce health and health care inequities (Thorndike et al., 2023). In California, the Medi-Cal program through the Department of Health Care Services is addressing health disparities through their CalAIM Sect. 1115 waiver by paying directly for services such as enhanced care management for Medi-Cal beneficiaries with high needs, community supports to help homeless beneficiaries secure and maintain housing, and targeted services for those who are justice involved prior to release (Department of Health Care Services. Medi-Cal Transformation Initatives (https://www.dhcs.ca.gov/CalAIM/Pages/Initiatives.aspx).

Advantages inherent in these approaches are that they propose solutions that are supported by regulatory agencies with sustainable funding streams. These approaches have disadvantages as well. Although each of the efforts identified above describe extensive community engagement, they are essentially top down and health care centered approaches to addressing social determinants of health. In contrast, contracting with community-based organizations to both assess community needs and to implement community-based interventions has the benefit of providing a community-centered approach that involves community members in identifying the underlying issues and their solutions. Investing more directly in communities can help to build community capacity through empowerment, normalizing help-seeking, and building connections. Thus, this approach may provide a framework for addressing health equity more generally in the community and in partnership with health systems. These approaches may be further bolstered by trauma-informed practices that destigmatize conditions (e.g., mental health problems or medical conditions) and situations (e.g., homelessness) associated with higher service needs and promote a sense of belonging and cohesiveness conducive to longer term health.

This study had some limitations. The participating partnerships, and their community impact, are context specific. The capacity-building efforts by the partnerships in this initiative may not translate to other partnerships or communities. Our goal was to ask quantitatively based questions of partnership staff that reflected their input during prior surveys and interviews. Therefore, our measurement is not broadly meant to assess all facets of capacity building nor the most essential elements of capacity building for other types of projects. In addition, our response rate for the partnership surveys was low at 25%. These limitations are offset by data obtained from a geographically large (~ 4000 square miles) and populous county, which exceeds the population of 42 states in the US; and an initiative that encompassed different strategies specifically tailored to the needs of diverse subcommunities.

We purposefully sampled community members who had some connection with the initiative that represented the diversity of community members’ cultural backgrounds and a range of capacity building strategies. However, only three of the partnerships participated in data collection. The pandemic delayed the focus groups, which were held in the final quarter of the initiative. This timing allowed evaluators to assess the initiative’s impact on community members' well-being after a shared trauma. However, it also coincided with a period of uncertainty as the partnerships prepared for the end of grant funding by facilitating warm handoffs of participants to other organizations and transitioning community ambassadors to new roles when possible. The three partnerships that had the capacity to support focus group recruitment are representative of the initiative and regionality of Los Angeles County, ranging from the urban Central Los Angeles (Watts) and Long Beach communities and more rural communities within the Antelope Valley. Focus groups were conducted in English, Spanish, and Khmer and included participants from diverse underrepresented populations and age groups.

Despite these limitations, investing in regional partnerships appears to have been an effective approach to support building community capacity to address trauma. The Los Angeles County Department of Mental Health is currently funding a second wave of forty smaller regional partnerships that will pursue capacity building with more targeted interventions. This new effort provides an opportunity to formalize the process of community needs assessment and subsequent capacity building using a community-engaged implementation framework that supports the goal of health equity for all (Schlechter et al., 2021). Tools such as the Implementation Research Logic Model (Smith et al., 2020) and the Inventory of Factors Affecting Successful Implementation and Sustainment (Becker at al., 2023), supported by implementation coaching and implementation learning collaboratives will help to provide scaffolding and structure to identifying community needs and implementation strategies, providing continuous feedback, and assessing outcomes. A goal of future work will be to both support successful capacity building efforts within current initiatives and to identify generalizable strategies to support similar efforts in varied contexts.

## Conclusion

Overall, this study demonstrates the potential for community-based collaborative partnerships to build community resilience to trauma through outreach and engagement with community members, linkages of community members to existing services, and provision of services for specific target populations. Such partnerships hold enormous potential in promoting health equity in communities that are especially vulnerable to exposure to trauma and their social and psychological consequences.

## Appendix

See Table

**Table 5:** Distribution of 101,370 Successful Linkages among 12,662 Unique Community Members to Resources and Services from January 2018 through July 2023

Type of linkage	Number of linkages	Distribution of linkages (%)	Examples of linkages
Anger management	866	0.9	Anger management training
Basic Needs	3829	3.8	Personal hygiene or household products or diapers
COVID	1373	1.4	COVID-19 education, testing, vaccination
Case management	2232	2.2	Individual support and coordination of services
Childcare	1006	1.0	Provision or financial support for childcare
Clothing	8818	8.7	Clothing distribution
Domestic violence	1022	1.0	Counseling, educational programs, safe housing, trauma support
Education	9718	9.6	Civics classes/immigration support, child development/parenting classes, education supports
Employment	2633	2.6	Employment opportunities and supports
Family	3948	3.9	Individualized family supports
Financial	937	0.9	Financial education, financial supports
Food	43,017	42.4	Food distribution, financial support to local restaurants
Health care	6349	6.3	Physical, dental, and behavioral health services
Housing	6727	6.6	Emergency housing, referrals to housing, rental support
Legal	997	1.0	Legal support
Other	1482	1.5	Donations, gift cards, services to support well-being
Support group	2772	2.7	Arts, peer or social support, knitting circle, storytelling, youth activities
Technology	924	0.9	Digital education and technology acquisition
Transportation	2720	2.7	Provision or financial support for transportation

5.
